# Coronary Pressure Measurement Based Decision Making for Percutaneous Coronary Intervention

**DOI:** 10.2174/157340309789317832

**Published:** 2009-11

**Authors:** Kohichiro Iwasaki, Shozo Kusachi

**Affiliations:** 1 Department of Cardiology, Okayama Central Hospital, Japan; 2 Department of Medical Technology, Okayama University Graduate School of Health Sciences, Japan

**Keywords:** Coronary pressure, fractional flow reserve, intermediate stenosis, tandem lesions, left main coronary artery, collateral blood flow.

## Abstract

The fractional flow reserve (FFR) is a simple, reliable, and reproducible physiologic index of lesion severity. In patients with intermediate stenosis, FFR≥0.75 can be used to safely defer percutaneous coronary intervention (PCI), and patients with FFR≥0.75 have a very low cardiac event rate. Coronary pressure measurement can determine which lesion should be treated with PCI in patients with tandem lesions, and PCI on the basis of FFR has been demonstrated to result in an acceptably low repeat PCI rate. FFR can identify patients with equivocal left main coronary artery disease who benefit from coronary bypass surgery. Coronary pressure measurement distinguishes patients with an abrupt pressure drop pattern from those with a gradual pressure drop pattern, and the former group of patients benefit from PCI. Coronary pressure measurement is clinically useful in evaluating sufficient recruitable coronary collateral blood flow for prevention of ischemia, which affects future cardiac events. FFR is useful for the prediction of restenosis after PCI. As an end-point of PCI, FFR ≥0.95 and ≥0.90 would be appropriate for coronary stenting and coronary angioplasty, respectively. In summary, if you encounter a coronary stenosis in doubt you should measure pressure rather than dilate it.

## INTRODUCTION

Percutaneous coronary intervention (PCI) is often performed without objective evidence of myocardial ischemia. Usually, a percent diameter stenosis of ≥50% as determined by quantitative coronary angiography is regarded to be significant stenosis [1]. However, necropsy and intravascular ultrasound studies demonstrate that coronary lesions are often highly complex, exhibiting markedly distorted or eccentric luminal shapes [2-4]. This complex morphology impairs the ability of coronary angiography to accurately depict coronary anatomy. The problem of coronary artery remodeling and the validity of a normal reference segment further limit the ability of coronary angiography [5]. Thus, coronary angiography has considerable limitations in evaluating coronary artery stenosis. 

The fractional flow reserve (FFR) is a simple, reliable, and reproducible physiologic index of lesion severity [6]. Several studies demonstrate that the FFR is strongly related to inducible myocardial ischemia established by rigorous comparisons to different clinical stress testing modalities [6-9]. Based on these considerations, FFR can be useful for evaluating coronary artery stenosis in various pathological conditions of coronary artery disease.

In this review we provide a short overview of the background of FFR and focus on its clinical application, both for diagnostic and therapeutic catheterization.

## CONCEPT AND CHARACTERISTICS OF FFR

**Fig. (1). Concept of FFR.**
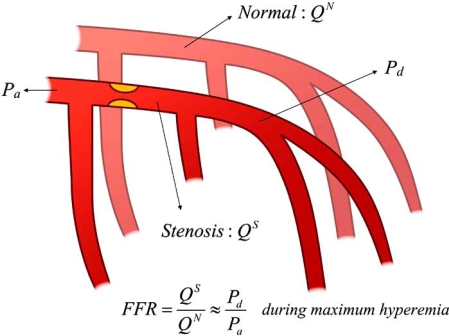
Q^S^: maximum myocardial blood flow in the presence of a stenosis Q^N^: normal maximum myocardial blood flow Pa: mean aortic pressure Pd: hyperemic distal coronary pressure

FFR is defined as maximum myocardial blood flow in the presence of a stenosis divided by the theoretical maximum blood flow in the same region in the absence of stenosis (Fig. **1**). This index represents the fraction of normal maximum blood flow that is achievable despite the presence of the epicardial coronary stenosis [6, 7, 10].

FFR= Q^S^/ Q^N^

Q^S^: maximum myocardial blood flow in the presence of a stenosis

Q^N^: normal maximum myocardial blood flow

Q^N^=(Pa-Pv)/R

Pa: mean aortic pressure, Pv: mean central venous pressure

R: myocardial resistance at maximum vasodilation

Q^S^=(Pd-Pv)/R

Pd: hyperemic distal coronary pressure

FFR=Pd-Pv/Pa-Pv

Because generally, central venous pressure is close to zero, this equation can be simplified to,

FFR=Pd/Pa

The advantages of FFR are as follows; 1) it has an uniform normal value of 1.0 for every patient and every coronary artery, 2) it’s independent of changes in blood pressure, heart rate, and contractility, 3) it has a clear cut-off value of 0.75 to discriminate significant from non-significant stenosis, and 4) it needs no control coronary artery, and 5) it accounts for collateral blood flow.

Regarding cut-off value of FFR, several studies have convincingly demonstrated that FFR of 0.75 accurately discriminates lesions with inducible reversible ischemia from those without inducible reversible ischemia [6-9]. An FFR <0.75 identified coronary stenosis in patients with inducible myocardial ischemia with high sensitivity (88%), specificity (100%), positive predictive value (100%), and overall accuracy (93%) [6].

## TECHNICAL ASPECTS OF CORONARY PRESSURE MEASUREMENT

**Fig. (2):**
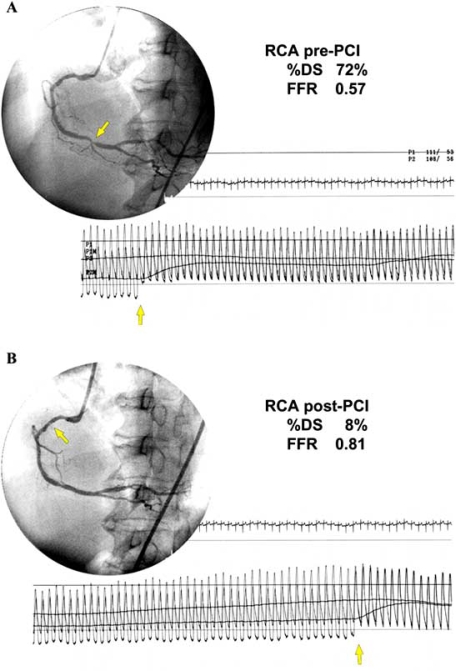
Usefulness of pull back pressure tracing. **A.** A 65-year-old woman had a 72% stenosis in the distal segment of the right coronary artery and FFR was 0.57. **B.** After coronary stenting percent diameter stenosis improved to 8% but FFR improved only to 0.81. Pull back curve showed that there was no pressure gradient at the stented segment and pressure gradient was found at the proximal portion of the right coronary artery, to which we performed coronary angioplasty three months earlier.

A pressure guide wire is zeroed, calibrated and advanced through the catheter into the coronary artery and positioned as distally as possible. Maximal hyperemia is then induced by intracoronary or intravenous continuous infusion of hyperemic agents (papaverine, adenosine, and adenosine triphosphate, Table **1**). FFR is then calculated at the maximum hyperemia from the simultaneously recorded aortic (Pa) and distal coronary pressure (Pd) by the ratio of Pd/Pa. Thereafter the pullback pressure tracing from a point as distal as possible to the proximal part of the coronary artery under steady-state maximum hyperemia is obtained and recorded at paper speed of 5mm/sec. The pullback pressure tracing is performed twice to confirm the accuracy of FFR obtained. Continuous intravenous infusion is the best method to obtain stable maximum hyperemia and provides an excellent pull-back curve. Pullback curve at maximum hyperemia is the most accurate, most convincing, and most reliable way to study the functional status of every part of a coronary artery (Fig. **2**) [11, 12].

## FFR IN MICROVASCULAR DISEASE

FFR has one theoretical limitation, which is microvascular disease. In patients with microvascular disease such as myocardial infarction, left ventricular hypertrophy, and diabetes mellitus, epicardial blood flow will not be as high as it would be in the case of normal microvascular function, which leads to overestimated FFR values. However, from a practical standpoint, FFR will still indicate to what extent the epicardial stenosis contributes to the limitation of the maximal perfusion in a given patient and to what extent the removal of the epicardial stenosis would lead to the improvement regardless of microvascular response.

In fact, there are several studies regarding the usefulness of FFR after myocardial infarction. De Bruyne *et al. *performed myocardial perfusion single photon emission scintigraphy (SPECT) and FFR measurement in 57 patients with myocardial infarction ≥6 days earlier [13]. The sensitivity and specificity of the 0.75 values of FFR to detect flow maldistribution at SPECT imaging were 82% and 87%, respectively. Further, the concordance between the FFR and SPECT imaging was 85%. Another study examined 48 patients 3.7±1.3 days after myocardial infarction [14]. The sensitivity, specificity, positive and negative predictive value, and concordance of FFR ≤0.75 for detecting true reversibility on SPECT were 88%, 93%, 88%, 93%, and 92%, respectively. The optimal FFR value for discriminating inducible ischemia was 0.78. Therefore, FFR of the infarct-related artery accurately identifies reversibility on non-invasive imaging early after myocardial infarction. 

## CLINICAL APPLICATION OF CORONARY PRESSURE MEASUREMENT (TABLE 2)

### 1. Intermediate Stenosis

Fig. (**3**) shows the relationship between percent diameter stenosis and FFR in our 417 patients with intermediate coronary stenosis, which is defined as percent diameter stenosis (% DS) between 40% and 70%. Our results show that considerable number of patients with intermediate stenosis had functionally non-significant stenosis. Our results also showed that coronary angiography could not discriminate between significant and non-significant stenosis in intermediate stenotic lesions. There was a large overlap of %DS between patients with normal FFR and those with abnormal FFR and the relationship between %DS and FFR in all patients was modest. Similar to our results, a study measured FFR in 83 patients with intermediate stenosis and compared the results with visual assessment and quantitative coronary angiography (QCA) of angiography. They found that neither visual assessment by experienced interventional cardiologist nor QCA could accurately predict the significance of intermediate stenosis [15]. These results indicate that FFR is clinically useful to evaluate the severity of intermediate stenosis accurately.

In patients with normal FFR the coronary event rate is lower with medical therapy as compared with coronary intervention. Eight previous studies, which included 24 to 150 patients, reported a coronary event rate, defined as death, myocardial infarction, PCI, bypass surgery, of 8% to 21% (mean 11%) during follow-up period of 11 to 29 (mean 16.3) months, which means the annual event rate was 5.7% [16-23]. They also reported the annual hard event rate, defined as death and myocardial infarction, of 1% per year. This very low hard event rate is comparable with that of patients with normal stress myocardial perfusion imaging, which is reported to be 0.6-1.0% per year [24-26]. Recently Pijls *et al. *reported the five-year follow-up data of the DEFER (Deferral versus Performance of PTCA in patients without Documented Ischemia) study [27]. Event-free survival was not different between patients with normal FFR and deferred PCI and those with normal FFR and performed PCI (80% and 73%, NS, respectively). Composite rate of cardiac death and myocardial infarction was 3.3% and 7.9%, respectively (NS).

Taken together, in patients with intermediate stenosis FFR ≥0.75 can be used to safely defer PCI, and that patients with FFR ≥0.75 have a very low cardiac event rate.

### 2. Tandem Lesions

In tandem lesions, evaluation of the severity of each lesion by coronary angiography is often difficult because of the complicated hydromechanics across tandem lesions. It is especially difficult to determine the severity of the tandem lesions when one or both lesions show intermediate stenosis [28]. Several noninvasive methods, such as stress myocardial perfusion imaging, are performed to evaluate the myocardial ischemia. However, these tests cannot determine which lesion of the two is responsible for reversible ischemia. Simple and reliable methods for evaluating the functional severity of the lesions are essential for decision making for PCI, especially in patients with equivocal tandem lesions. An animal study demonstrated that FFR of each lesion in a tandem lesion case could not be calculated using the equation for isolated stenosis applied to each lesion separately [29]. Another clinical study also indicated that the FFR of each stenosis would have been significantly overestimated without accounting for stenosis interaction [30]. 

To resolve this issue, Hirota *et al. *measured coronary pressure in 72 patients with tandem lesions [31]. For patients with FFR ≥0.75 across the tandem lesions they deferred PCI. When FFR was <0.75 across the tandem lesions, they performed PCI for the angiographically more severe stenosis. If FFR after PCI for the first lesion was ≥0.75, they deferred PCI for the second lesion. If FFR after PCI for the first lesion was <0.75, they performed PCI for the second lesion. As a result they deferred PCI for 26 patients (36.1%), performed PCI for one lesion in 19 patients (26.4%), and PCI for both lesions in 27 patients (37.5%). Among patients in whom PCI was deferred, only two patients (7.7%) required PCI during the follow-up period. This rate was not significantly different from that in the 46 patients who underwent PCI for one or two lesions (six patients, 13.0%). Thus this study showed that in patients with tandem lesions, FFR could clearly discriminate functionally significant stenosis and safely deferred PCI in those with normal FFR.

### 3. Equivocal Left Main Coronary Artery Disease

Assessment of left main coronary artery (LMCA) disease by coronary angiography is often suboptimal [32, 33]. It is often difficult to make the decision of whether coronary revascularization for equivocal LMCA disease is required or not. Pathological studies demonstrated that atherosclerosis diffusely involves the coronary arteries and recent intravascular ultrasound (IVUS) studies confirmed this finding *in vivo* [3, 34]. Intravascular ultrasound studies also found that coronary remodeling frequently occurs, which prevents the narrowing of the coronary arterial lumen through compensatory enlargement of the coronary artery [5]. These findings indicate that a true reference segment cannot be identified by coronary angiography. In fact, Cameron *et al. *studied the reliability of angiographic assessment of LMCA stenosis [33]. They reviewed 106 coronary angiograms and found that there was only 41% to 59% agreement on the severity of the lesion among three groups of angiographers. Another study performed intravascular ultrasound study in 60 patients [35]. While significant LMCA stenosis was present in 2 patients IVUS demonstrated plaques in 27 of 60 LMCAs, six of them in patients with normal angiograms. When the LMCA lesion is immeasurably short, diffusely diseased, or obscured by overlapping vessels, determination of severity of the LMCA by angiography is especially difficult. Therefore, angiographic assessment is not reliable for the accurate evaluation of LMCA stenosis.

Furthermore, if an LMCA stenosis is not functionally significant, a bypass graft will be occluded and unnecessary bypass surgery will be performed. There is a study that followed 122 patients with moderate (≥50% diameter stenosis) LMCA stenosis for one year [36]. The lesion site minimum lumen diameter by quantitative coronary angiography correlated moderately with IVUS (r=0.364) and they found that one-year event rate was only 14%, and only four patients died. This study indicates that most patients with moderate LMCA stenosis do not benefit from CABG and that these patients may not have functionally significant LMCA stenosis. 

If LMCA stenosis is not functionally significant and functionally significant coronary stenosis exists in the other part of coronary artery, PCI for this lesion should be the appropriate treatment. LMCA stenosis has been known to be frequently associated with coronary stenosis in the other part of coronary artery [37]. Non-invasive tests, such as myocardial perfusion imaging, often fail to differentiate between ischemia caused by LMCA stenosis and that caused by coronary stenosis located in the other part of the coronary artery.

Most of these problems in evaluating LMCA stenosis can be solved by using coronary pressure measurement. There are several studies which selected treatment strategy based on FFR results in patients with equivocal LMCA disease. Suemaru *et al. *studied 15 patients with equivocal LMCA stenosis [38]. Eight patients with FFR≥0.75 received medical therapy and seven patients with FFR <0.75 underwent CABG. During the follow-up period of 32.5±9.7 months, no adverse cardiac event occurred in patients with medical therapy. Two patients with CABG were hospitalized with congestive heart failure. Bech *et al. *measured FFR in 54 patients with equivocal LMCA stenosis and assigned 24 patients with FFR≥0.75 to medical treatment and 30 patients with FFR <0.75 to CABG [39]. Event-free survival at three years was not significantly different between the medical group and surgical group (76% *vs*. 83%, respectively). In medical group two patients underwent coronary angioplasty and three patients underwent CABG. These results clearly show that FFR can accurately evaluate the severity of LMCA stenosis. Careful follow-up of patients with medical therapy is very important. Rupture of coronary plaque in the LMCA followed by thrombus formation will likely result in a catastrophic event [40, 41]. Therefore, patients with medical therapy should be carefully observed with intensive medical therapy, including statin and ACE inhibitors.

As for coronary pressure measurement of LMCA stenosis, the position of the catheter is critical for the accurate assessment. The tip of the catheter should not be engaged in LMCA but rather should be slightly removed from the ostium of LMCA, which enables the precise measurement of the proximal coronary pressure of the LMCA stenosis. Then the pressure wire is gradually pulled back under fluoroscopy. Caution should be made to not allow deep engagement of the tip of the catheter into the LMCA during gradual pull-back of pressure wire. These careful coronary pressure measurements provide precise FFR measurement across the LMCA lesion.

Taken together, coronary pressure measurement can identify patients with equivocal LMCA disease who benefit from coronary bypass surgery.

### 4. Diffuse Coronary Artery Disease

Atherosclerosis usually involves the entire coronary artery tree to various extents [42, 43]. Diffuse atherosclerotic involvement of coronary arteries is one of the major factors affecting morbidity and mortality in patients with coronary artery disease [44, 45]. Thus detection and evaluation of the physiological significance of diffuse coronary artery disease have important therapeutic implications.

Coronary angiography again has some limitations for evaluating diffuse stenosis of the coronary arteries [28, 46, 47]. Current quantitative coronary angiographic measurement of percent diameter stenosis does not account for the true normal coronary arterial size because only the lumen of the reference segment is used for comparison [47]. The true size of an artery is often obscured because of coronary arterial remodeling [5]. Thus interpretations of coronary angiography severely underestimate mild or diffuse coronary atherosclerosis and overestimate the severity of stenosis with >50% diameter narrowing [44, 48]. Many studies have shown that visual interpretation of coronary angiography severely underestimates mild or diffuse coronary artery disease [28, 46, 47]. Seiler *et al. *showed that in patients with even mild segmental stenosis, the luminal diameters of coronary arteries over a broad region are 30-50% smaller than they should be for the regional mass served [49, 50]. Thus, coronary angiography is not a reliable method to determine the severity of diffuse coronary artery disease.

The frequency and fluid dynamic severity of diffuse coronary artery narrowing in patients with coronary artery disease have not been previously described. Coronary flow reserve measured distally by intracoronary Doppler or pressure guide wires is lower than that measured proximally and correlates better with stress perfusion defects than with proximal measurements of coronary flow reserve [7, 51, 52]. A study examined 1001 patients with documented coronary artery disease by positron emission tomography [53]. The study showed that 43% of patients with or without segmental perfusion defects demonstrated a graded, longitudinal, base-to-apex myocardial perfusion gradient, indicating dynamically significant diffuse coronary artery disease.

**Fig. (4). Abrupt pressure drop pattern.**
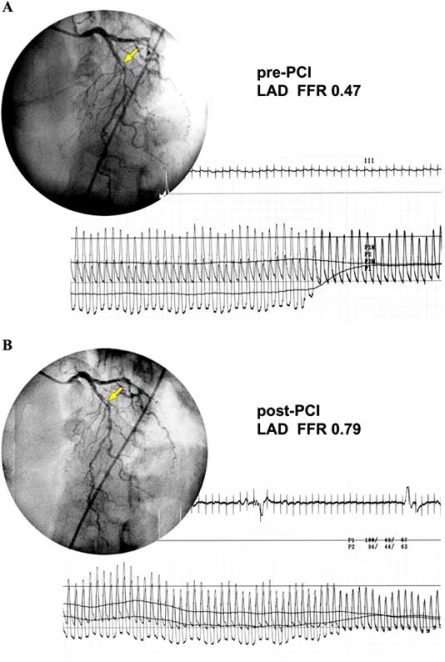
Pull back pressure tracing in a patient with an abrupt pressure drop pattern before (**A**) and after (**B**) treatment with PCI. An abrupt drop of coronary pressure was observed and FFR was 0.47. Six months after PCI, the pressure difference was reduced and FFR improved to 0.79.

**Fig. (5). Gradual pressure drop pattern.**
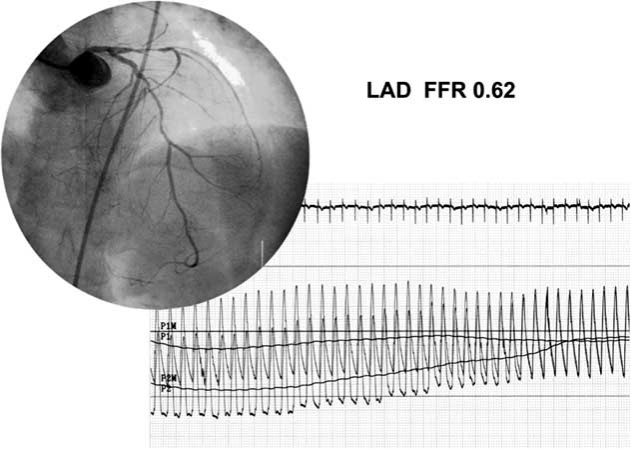
Pull back pressure tracing in a patients with a gradual pressure drop pattern. A gradual drop of coronary pressure was observed and FFR was 0.62.

Pijls reported two patients with two different patterns of coronary pullback pressure in angiographically diffuse coronary artery stenosis [54]. One patient showed a local pressure drop pattern and the other showed a gradual pressure drop pattern. We measured coronary pressure of the left anterior descending coronary artery in 83 patients with diffuse coronary artery disease. The abrupt pressure drop pattern was observed in 47 patients (57%), while the remaining 36 patients (43%) showed the gradual pressure drop pattern (Figs. **4**, **5**). Patients with an abrupt pressure drop pattern would benefit from localized therapy, which means PCI. CABG for gradual pressure drop lesions may provide some positive effects through the elevation of coronary perfusion pressure to the peripheral coronary artery tree.

Taken together, coronary pressure measurement can distinguish patients with an abrupt pressure drop pattern from those with a gradual pressure drop pattern, and the former group of patients would benefit from PCI.

### 5. Multivessel Disease

In multivessel disease accurate lesion selection for PCI is important. Some noninvasive studies demonstrated that SPECT fails to correctly indicate all ischemic area in 90% of patients. In one third of such patients, no perfusion defect was present because of balanced ischemia [55].

There is a study that measured FFR in 102 patients with multivessel disease, in whom PCI of at least two vessels was contemplated [56]. In all of them at least one vessel was treated by PCI, whereas at least one other vessel was deferred based on an FFR >0.75. No death occurred during the follow-up. The rate of major adverse cardiac event was not significantly different between treated and deferred arteries (p=0.64). Similarly, another study examined 137 patients with multivessel disease [57]. In 57 patients FFR measurement of all vessels were performed and PCI of stenoses with FFR <0.75 was performed (FFR-PCI group). The remaining 80 patients underwent PCI by visual estimation of the stenoses (conventional PCI group). The average number of vessels per patient that underwent PCI was greater in the conventional PCI group than in the FFR-PCI group (2.27±0.50 *vs*. 1.12±0.30 vessels, p<0.001). The 30-month event-free survival rate was significantly higher in the FFR-PCI group than in the conventional PCI group (89% *vs*. 59%, p<0.01). Thus, these studies suggest that in patients with multivessel disease, PCI of hemodynamically non-significant stensoses can be safely deferred, and FFR measurement is a cost-effective method. Currently the Fractional Flow Reserve versus Angiography for Multivessel Evaluation (FAME) study is underway to examine the role of measuring FFR in patients undergoing multivessel PCI [58].

### 6. Collateral Blood Flow

Collateral circulation potentially offers an important alternative source of blood supply when the original vessel fails to provide sufficient blood [59, 60]. There have been controversial results regarding the cardioprotective effects of angiographically present collaterals. Much of the argument is likely attributed to the blunt method of gauging human coronary collaterals by coronary angiography. Coronary angiography has been reported to be not a reliable method to evaluate collateral blood flow [28]. Recent studies which directly compare the angiographic scoring system and quantitative functional measures of collateral supply, such as pressure- or Doppler-derived collateral flow indexes, found only a very weak correlation, or none at all [61-63]. In contrast experimental and clinical studies, which measured coronary wedge pressure during coronary occlusion and directly determined collateral blood flow, demonstrated the close relationship between coronary wedge pressure and collateral flow [61, 64, 65]. Furthermore, a close relation between coronary wedge pressure and myocardial perfusion by positron emission tomography and ^99m^Tc-sestamibi has been reported [66, 67]. These studies support that the coronary wedge pressure measured by a pressure guide wire is reliable and valid for the assessment of coronary collateral flow. Angiographic collateral quantitation has been performed to look for spontaneously visible instead of recruitable collaterals, the latter of which appear in response to occlusion of the collateral receiving vessel and reflect collateral supply more comprehensively than the former.

During balloon inflation, coronary wedge pressure (Pw) is continuously recorded with the pressure guide wire. Aortic pressure (Pa) is also recorded through the guiding catheter. Recruitable collateral blood flow (Qc/Q^N^) is expressed as a fraction of normal maximum myocardial perfusion (Q^N^): Qc/Q^N^ = (Pw-Pv)/(Pa-Pv), where Pv is central venous pressure. 

Regarding the cutoff value that indicates sufficient collateral blood flow to prevent ischemia during coronary occlusion, a Qc/Q^N^ value of 0.20 to 0.30 has been reported by different methods [68, 69]. A physiological study demonstrated that one-quarter of the resting coronary flow meets the basal metabolic requirements for maintaining the metabolic integrity of the cardiac cells [70].

Kamikawa *et al. *measured coronary pressure during coronary occlusion with PCI in 119 patients with left anterior descending coronary artery stenosis [71]. A significant relationship between Qc/Q^N^ and maximum ST elevation was observed (r=-0.455, p<0.0001). Also Qc/Q^N^ was significantly correlated with the sum of the maximum ST elevation in leads V2-V4 (r=-0.477, p<0.0001). Thus, recruitable collateral blood flow determined by coronary pressure measurement correlated with myocardial ischemia estimated by electrocardiographic changes during coronary occlusion.

Recent studies demonstrate a beneficial impact of well-developed collaterals on the occurrence of future major cardiac ischemic events. Billinger *et al. *studied 403 patients with stable coronary artery disease undergoing coronary angioplasty and calculated collateral flow index using intracoronary pressure or Doppler guidewires [72]. During follow-up period of 94±56 weeks, major cardiac ischemic event rate was 2.2% in patients with well developed collateral blood flow compared with 9.0% in those with poorly developed collateral blood flow (p=0.01). Similarly, another study reported the beneficial effect of recruitable collaterals [73]. They measured coronary pressure in 739 patients with stable coronary artery disease and calculated collateral flow index. Cumulative 10-year survival rate was 89% and 71% in patients with well-grown collateral group and poorly developed collateral group, respectively (p=0.0395). Thus, well-functioning coronary collateral circulation saves lives in patients with stable coronary artery disease and accurate determination of collateral blood flow is important.

Taken together, coronary pressure measurement with use of cut off value around 0.30 is clinically useful in evaluating sufficient recruitable coronary collateral blood flow for prevention of ischemia, which affects future cardiac events.

In summary, if you encounter a coronary stenosis in doubt you should measure pressure rather than dilate it.

### 7. End Point of PCI

Restenosis is a major limitation of PCI and even drug-eluting stents do not eliminate this phenomenon. The restenosis rate after coronary angioplasty is reported to be 30-50% and that of coronary stenting by bare-metal stent is 20-30%. A cardiac event occurs in -30% of patients after coronary angioplasty and ‒15% after coronary stenting [74-77]. Recent drug-eluting stent studies show a very low restenosis rate. However the restenosis rate is reported to be 5-10% and cardiac event occurs in ‒10% of patients [78, 79]. Many studies demonstrate that coronary angiography, including quantitative coronary angiography, is poor predictor of restenosis [80, 81]. Therefore, alternative approaches to assess lumen enlargement, such as intravascular ultrasound, have been advocated to better identify patients in whom restenosis is less likely [82, 83].

Usefulness of FFR for predicting clinical outcomes after coronary angioplasty has been reported [19]. A study examined 60 patients who underwent successful coronary angioplasty using FFR. Patients with FFR ≥0.90 and %DS ≤35% (optimal group) showed better event-free survival rate compared with those FFR <0.90 and/or %DS >35% (suboptimal group, 92±5% *vs*. 72±8% at six months, respectively, p=0.047). Repeat PTCA rate was 3.8% in the optimal group and 15.6% in the suboptimal group at six months after PTCA, which was not significantly different. Thus, in patients with a residual %DS ≤35% and FFR ≥0.90, clinical outcome up to 2 years was excellent.

Pijls *et al. *reported the usefulness of FFR to predict adverse events after coronary stenting [84]. They reported the adverse cardiac events of 750 patients who underwent coronary stenting. A significant inverse correlation was found between final FFR and occurrence of adverse events during follow-up. Also the correlation was significant for repeat target vessel revascularization. At 6 months follow-up, adverse cardiac rate was 4.9% and 6.2% in patients with FFR 0.96-1.00 and 0.91-0.95, respectively (p<0.001). According to multivariate analysis, FFR after coronary stenting was a strong independent predictor of outcome at 6 months. Another study enrolled 119 patients who underwent coronary stent implantation and studied the predictors of cardiac events [85]. They found that final FFR <0.95 and reduced left ventricular function remained as significant independent predictors in a multivariate analysis. 

Similarly Fearon *et al. *measured FFR and made a comparison with intravascular ultrasound findings in 84 patients with stent implantation. They found an optimal FFR cutoff point of 0.96 by receiver operator characteristic analysis [86]. There is also a study that compared QCA, IVUS, and FFR to assess optimal stent deployment in 30 patients who underwent coil-stent implantation [87]. The study found that 91% of FFR and IVUS findings yielded concordant results but QCA showed a low concordance rate with FFR and IVUS. As for slotted-tube stents substantial concordance between FFR and IVUS findings has been reported [88].

Taken together, FFR can be used as a good indicator of optimal PCI results. As an end point of PCI, FFR ≥0.90 and ≥0.95 would be appropriate for coronary angioplasty and coronary stenting, respectively.

## Figures and Tables

**Fig. (3):**
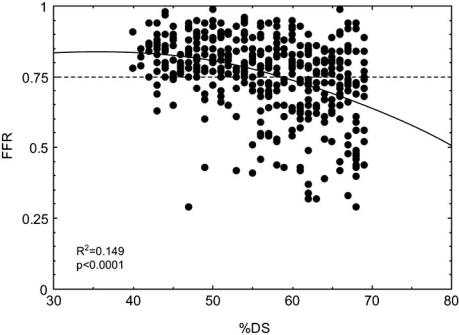
The relationship between percent diameter stenosis (%DS) and fractional flow reserve (FFR).

**Table 1:** Pharmacological Agents for Coronary Hyperemia

	Papaverine	Adenosine	Adenosine Triphosphate
Increase in CBF	4-6 times	4-6 times	4-6 times
Half-life	60-90sec	<20sec	<20sec
Plateau ic	30-60sec	5-10sec	5-10sec
iv	—	<1-2min	<1-2min
Dose			
ic RCA	10mg	30µg	30µg
LCA	15-20mg	40-80µg	40-80µg
iv	—	140-160µg/kg/min	140-160µg/kg/min
Side Effect	QT prolongation	angina-like chest pain	angina-like chest pain
	VT/Vf	BP decrease by 10-15%	BP decrease by 10-15%

**Abbreviations:** BP; blood pressure, CBF; coronary blood flow, ic; intracoronary, iv; intravenous, LCA; left coronary artery, RCA; right coronary artery, VT; ventricular tachycardia, Vf; ventricular fibrillation.

**Table 2:** Clinical Application of Coronary Pressure Measurement

1.	Intermediate stenosis
2.	Tandem lesions
3.	Equivocal left main coronary artery disease
4.	Diffuse coronary artery disease
5.	Multivessel disease
6.	Assessment of collateral blood flow
7.	Determining end point of PCI
